# Resveratrol protects intestinal epithelial cells against radiation-induced damage by promoting autophagy and inhibiting apoptosis through SIRT1 activation

**DOI:** 10.1093/jrr/rrab035

**Published:** 2021-04-29

**Authors:** Haoren Qin, Heng Zhang, Xipeng Zhang, Shiwu Zhang, Siwei Zhu, Hui Wang

**Affiliations:** Tianjin University of Traditional Chinese Medicine, Tianjin 300193, China; Tianjin Union Medical Center of Nankai University, Department of Oncology, Tianjin 300121, China; Tianjin Union Medical Center of Nankai University, Department of Colorectal Surgery, Tianjin 300121, China; Tianjin Union Medical Center of Nankai University, Department of Pathology, Tianjin 300121, China; Tianjin Union Medical Center of Nankai University, Department of Oncology, Tianjin 300121, China; Tianjin Union Medical Center of Nankai University, Department of Oncology, Tianjin 300121, China

**Keywords:** autophagy, ionizing radiation, resveratrol, sirtuin 1 (SIRT1), radiation protection

## Abstract

Intrinsic autophagy is important for the maintenance of intestinal homeostasis and intestinal regeneration. Ionizing radiation suppresses intrinsic autophagy and reduces damage-induced regeneration in the intestine, resulting in intestinal injury. Resveratrol, a sirtuin 1 (SIRT1) agonist, promotes autophagy and exerts radioprotective effect. In this study, the protective effect of resveratrol against radiation-induced intestinal injury and its potential mechanism were investigated. Intestinal epithelial cells (IEC-6) were exposed to 10 Gy ionizing radiation and resveratrol (0.1–40.0 μM). Cell viability was investigated using Cell Counting Kit 8 (CCK8), apoptosis was observed by Annexin V-fluorescein isothiocyanate/propidium iodide (PI) staining and flow cytometry, and the expression of apoptotic and autophagic proteins was determined by western blotting. Resveratrol exerted a high toxicity against IEC-6 cells, but at low concentrations, it inhibited ionizing radiation-induced apoptosis. Resveratrol increased SIRT1 expression after irradiation and inhibited ionizing radiation-induced p53 acetylation and pro-apoptotic protein, Bax, expression. Furthermore, resveratrol promoted autophagy via the phosphoinositide 3-kinase (PI3K)/AKT/mammalian target of rapamycin (mTOR) pathway, thereby protecting IEC-6 cells against radiation-induced damage. These results suggest that resveratrol reduces radiation-induced IEC-6 cell damage by inhibiting apoptosis and promoting autophagy via the activation of SIRT1, and that the PI3K/AKT/mTOR signaling pathway is involved in the induction of autophagy.

## INTRODUCTION

Radiotherapy plays an important role in the treatment of various tumors. However, irradiating cancerous regions causes unavoidable damage to the surrounding healthy tissue. Radiation enteritis is the most common complication following abdominal or pelvic radiotherapy; the main symptoms include abdominal pain, diarrhea, bleeding and tenesmus [1]. Severe cases of radiation enteritis can lead to bowel perforation and peritonitis, which can be life threatening [2]. To avoid radiation enteritis, radiologists limit each irradiation dose and the total irradiation dose to the tumor target area, resulting in underdosing to the target and poor tumor control. These factors have seriously affected the quality of life and prognosis of patients. At present, besides symptomatic treatments there are no effective clinical treatments for radiation enteritis. Thus, there is need for effective prevention and treatment measures for radiation enteritis.

Resveratrol (3,4′,5-trihydroxy-trans-stilbene), a naturally occurring polyphenolic compound, has multiple pharmacological effects including anti-oxidant, anti-aging, anti-inflammatory and anti-apoptotic effects [3,4]. Extensive research indicates that resveratrol is a potential radioprotective agent. Resveratrol can ameliorate irradiation-induced hepatic damage [5], hematopoietic stem cell injury [6] and mouse embryonic stem cell injury [7]. Sirtuin 1 (SIRT1) is a nicotinamide adenine dinucleotide-dependent protein deacetylase that can be activated by resveratrol [8]. Previous work has found that resveratrol also has a protective effect against radiation-induced intestinal injury, partially owing to the activation of SIRT1 [9]. In this study, we found that resveratrol promotes autophagy and inhibits apoptosis by activating SIRT1, thereby reducing radiation-induced damage.

Autophagy is a highly regulated, evolutionarily conserved, lysosome-mediated degradation process, which can eliminate and recycle damaged organelles and related macromolecules to maintain cellular homeostasis [10]. A previous study showed that intrinsic autophagy is active in normal intestinal stem cells (ISCs) at a steady state and this is important for supporting ISC physiological homeostasis and intestinal regeneration. Autophagy-deficient mice had significantly fewer ISCs and showed impaired ISC-dependent recovery after irradiation both *in vitro* and *in vivo* [11]. Related research has demonstrated that radiation exposure can significantly downregulate autophagy, accompanied by altered phosphoinositide 3-kinase (PI3K)/AKT/mammalian target of rapamycin (mTOR) signaling pathway in mice intestine and IEC-6 cells, resulting in structural and functional alterations of the gastrointestinal tract [12]. Therefore, modulating autophagy is a potential therapeutic strategy, especially as a previous study has reported that promoting autophagy protected the intestine against radiation-induced injury [13]. Resveratrol has been shown to induce autophagy in several diseases, thereby serving a beneficial role [14–16]. However, it is unknown whether resveratrol could attenuate ionizing radiation-induced damage in rat intestinal epithelial cells (IEC-6) by promoting autophagy. Thus, in this study, we evaluated the protective effect of resveratrol against radiation-induced injury in IEC-6 cells and explored the potential molecular mechanisms that coordinated these effects. The results provide theoretical support for the use of resveratrol in radiation enteritis treatment.

## METHODS

### Cell culture

IEC-6 cell line was purchased from the Research Facilities of the Peking Union Medical College Cell Bank. The cells were cultured in Dulbecco’s modified Eagle’s medium (Thermo Fisher Scientific, Waltham, MA, USA) supplemented with 10% fetal bovine serum (Gibco, Gaithersburg, MD, USA) and 1% penicillin–streptomycin (Gibco), and incubated at 37°C in a constant-temperature cell incubator containing 5% CO_2_.

### Ionizing radiation and cell treatments

The cells were exposed to 10 Gy ionizing radiation using 6 MeV high-energy X-rays generated by a linear accelerator (Varian Clinic 21ES; Varian Medical Systems, Crawley, UK) at a dose rate of 0.99 Gy/min. Resveratrol (R5010; Sigma-Aldrich, St. Louis, MO, USA) was dissolved in dimethyl sulfoxide (DMSO) and diluted with the culture medium for 24 h to obtain solutions of various concentrations for use in the treatment groups. The control and irradiation group cells were incubated in the culture medium containing an equal amount of DMSO. According to previous studies, the selective inhibitor Ex527 (E7034; Sigma-Aldrich) at a concentration of 10 μM exhibits an inhibitory effect on the deacetylation activity of SIRT1. Therefore, the cells were treated with 10 μM Ex527 to inhibit the deacetylation activity of SIRT1 [17,18].

### Cell Counting Kit 8 assay for the cytotoxicity of resveratrol

The cytotoxicity of resveratrol was evaluated using the Cell Counting Kit 8 (CCK8) assay. Briefly, IEC-6 cells were plated in 96-well plates at a density of 3000 cells/well, and five auxiliary wells were set for each group. After 24 h of incubation, the cells adhered to the wall. The culture medium was replaced with medium containing resveratrol at various concentrations, and the cells were incubated for 24 h at 37°C under 5% CO_2_.Thereafter, 10 μL of CCK8 reagent (#CA1210, Solarbio, Beijing, China) was added to each well and the plates were incubated for 2 h. Cell viability was determined by measuring the absorbance of the samples at 450 nm using a microplate reader (Bio-Rad, Hercules, CA, USA).

### Trypan blue dye exclusion assay for cell viability

The viability of cells after irradiation was assessed using the trypan blue dye exclusion assay. The cells were plated in six-well plates and irradiated when the cell density reached 70–80%. Each group was pretreated with resveratrol at the indicated concentrations 2 h before irradiation. After 24 h of incubation, the medium was transferred into a centrifuge tube. The attached cells were trypsinized, collected and washed with phosphate-buffered saline (PBS). The cells were then pelleted by centrifugation and the supernatant was discarded. The cell pellet was resuspended in normal medium, and 100 mL of cell suspension was mixed with 100 mL of trypan blue dye. A drop of the suspension was placed on a hemocytometer, and then live (unstained) and dead (stained) cells were quantified under a microscope.

### Annexin V-fluorescein isothiocyanate/propidium iodide staining for determining apoptosis by flow cytometry

The Annexin V-fluorescein isothiocyanate **(**FITC)/propidium iodide (PI) cell apoptosis kit (C1062L, Beyotime, Beijing, China) was used to quantify IEC-6 cell apoptosis according to the manufacturer’s instructions. Briefly, IEC-6 cells were harvested following the different treatments, and then washed twice with PBS. The cells were resuspended in the staining buffer provided in the kit, and the cell number was adjusted to 5–10 × 10^5^/mL. Thereafter, 1 mL of the cell suspension was mixed with 5 μL of Annexin V-FITC and 10 μL of PI. After 20 min of cultivation at room temperature, the mixture was analyzed using a FACScan flow cytometer (BD Biosciences, Franklin Lakes, NJ, USA).

### Western blotting

Cells were collected 24 h after irradiation and washed three times with PBS. The total protein was extracted using a lysis buffer containing a cocktail of protease inhibitors (Thermo Scientific, 87785). Equal amounts of proteins were separated by sodium dodecyl sulfate-polyacrylamide gel electrophoresis, transferred onto a polyvinylidene difluoride membrane, and blocked with 5% skimmed milk at room temperature for 2 h on a gentle shaker. The membranes were then incubated with primary antibodies overnight at 4°C. The primary antibodies were as follows: SIRT1 (9475S; Cell Signaling Technology, Danvers, MA, USA; 1:1000 dilution), mTOR (2983S; Cell Signaling Technology; 1:1000 dilution), LC3A/B (D3U4C) (12741S; Cell Signaling Technology; 1:1000 dilution), beclin-1 (11306–1-AP; Wuhan Biotechnology, Wuhan China; 1:2000 dilution), acetyl-p53 (Lys382) (2525S; Cell Signaling Technology; 1:500 dilution), and β-actin (3700S; Cell Signaling Technology; 1:3000). The membranes were washed three times with Tris-buffered saline/0.1% Tween-20 and incubated with an anti-rabbit IgG horseradish peroxidase-linked antibody (7074S; Cell Signaling Technology; 1:5000 dilution) for 2 h at room temperature. The proteins were detected using an enhanced chemiluminescence kit (Pierce Biotechnology Inc., Rockford, IL, USA) and quantified using the ChemiDoc imaging system (Bio-Rad) and Image-Pro Plus 6.0 software. β-Actin was used as the loading control.

### Statistical analyses

SPSS version 20 (IBM Corp., Armonk, NY, USA) was used for statistical analyses. Kolmogorov–Smirnov method was used to test the normal distribution of data. Data are expressed as mean ± standard deviation. An analysis of variance was used for comparisons among multiple groups. For post-hoc pairwise comparisons, the LSD test was used when variances were homogeneous, and Dunnett’s T3 test was used when variances were not homogeneous. Descriptive statistics were performed and figures were created using GraphPad Prism 5.0 (GraphPad Software, La Jolla, CA, USA). All experiments were repeated at least three times. A significant difference was defined at *P* < 0.05.

## RESULTS

### Effect of resveratrol on the viability of IEC-6 cells

**Fig. 1.**
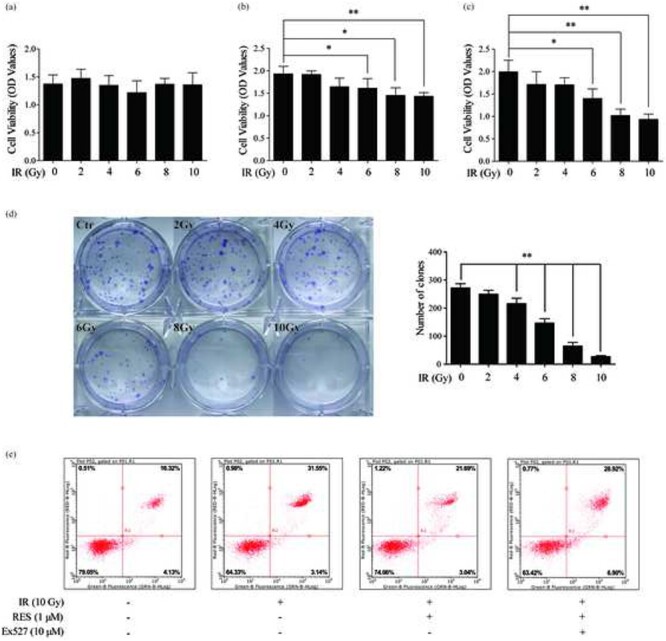
(A) Cell viability was measured using the CCK8 assay with IEC-6 cells incubated with RES for 24 h. (B) Cell viability was measured using the trypan blue dye exclusion assay with IEC-6 cells incubated with RES for 24 h after irradiation. (C) Apoptosis detected by flow cytometry after Annexin V-FITC (RED-B-HLog) and PI (GRN-B-HLog) staining. Data are expressed as mean ± standard deviation. ^*^P < 0.01 compared with the control group; ^#^*P* < 0.01 compared with the ionizing radiation group.

Resveratrol had no significant effect on cell viability at 0–5 μM concentrations. However, cell viability decreased at 10 μM, and obvious cytotoxicity was observed at higher concentrations (Fig. 1a). Thus, we believe that the non-toxic concentration range of resveratrol for IEC-6 cells is 0–5 μM, and this range was selected for the subsequent experiments.

### Resveratrol reduces radiation-induced damage to IEC-6 cells by inhibiting apoptosis

With the non-toxic concentration range of resveratrol, the trypan blue dye exclusion assay was used to determine the viability of IEC-6 cells at 24 h after irradiation. The cell viability of the groups pretreated with low concentrations (0.1–1 μM) of resveratrol was significantly higher than that of the irradiation group. However, resveratrol at higher concentrations (2–8 μM) had no significant effect on the viability of IEC-6 cells after irradiation (Fig. 1b).

To further verify the radioprotective effects of resveratrol, Annexin V FITC/PI staining was carried out. According to the results of the CCK8 assay, we chose 1 μM as the concentration of resveratrol. At 24 h after irradiation, the level of apoptosis of IEC-6 cells in the resveratrol pretreatment group was significantly lower than that in the irradiation group. However, when we stimulated the resveratrol pretreatment group with Ex527, a SIRT1 inhibitor, the apoptotic rate increased (Fig. 1c). These results show that resveratrol reduced radiation damage in IEC-6 cells by inhibiting apoptosis, and its mechanism may involve the activation of SIRT1.

### Resveratrol activates SIRT1 and inhibits p53 protein acetylation

**Fig. 2.**
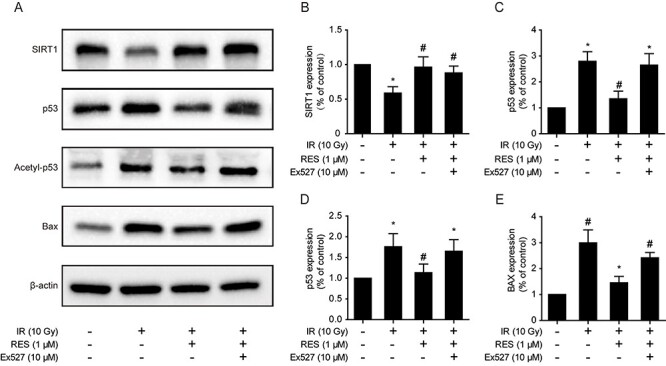
(A) Protein levels of SIRT1, p53, acetylated (acetyl)-p53 and Bax detected by western blotting. (B–E) The expression levels of SIRT1, p53, acetyl-p53 and Bax were quantified. Data are expressed as mean ± standard deviation. ^*^P < 0.01 compared with the control group; ^#^*P* < 0.001 compared with the IR group.

To assess whether the protective effect exerted by resveratrol on irradiated IEC-6 cells is attributed to SIRT1, we used western blotting to detect the expression of SIRT1 and apoptosis-related proteins (Fig. 2a). Our results showed that ionizing radiation downregulated the expression of SIRT1 and, after resveratrol pretreatment, SIRT1 expression was upregulated (Fig. 2b). Subsequently, we detected the level of p53, a SIRT1 substrate. The expression of p53 was significantly upregulated after irradiation, and resveratrol pretreatment downregulated p53 expression following irradiation; however, p53 expression increased in cells treated with Ex527 (Fig. 2c). Furthermore, we determined the level of acetylated p53, an active form of this protein, in our samples. p53 acetylation was significantly upregulated after irradiation and Ex527 treatment, whereas resveratrol pretreatment downregulated p53 acetylation following irradiation (Fig. 2d). We also detected the expression of the pro-apoptotic protein, Bax, a downstream effector of p53. Resveratrol pretreatment reduced the expression of Bax after irradiation, whereas SIRT1 inhibition with Ex527 reversed the effects induced by resveratrol (Fig. 2e).

### Resveratrol promotes autophagy via the SIRT1/mTOR pathway

**Fig. 3.**
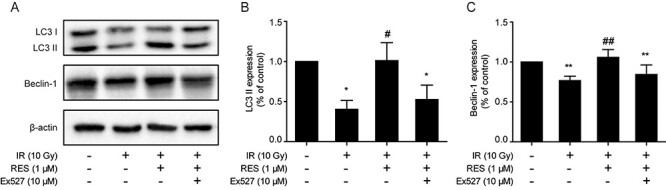
(A) Protein levels of LC3-I/II and beclin-1 detected by western blotting. Expression of (B) LC3-II and (C) beclin-1. Data are expressed as mean ± standard deviation. ^*^P < 0.01 compared with the Ctr group; ^**^P < 0.05 compared with the control group; ^#^*P* < 0.01 compared with the IR group; ^##^*P* < 0.05 compared with the IR group.

The expression of the autophagy proteins, LC3-II and beclin-1, in the ionizing radiation group was decreased compared with that in the control group (Fig. 3a). Resveratrol pretreatment increased the expression of LC3-II and beclin-1 (Fig. 3b and c), as well as increased the conversion of LC3-I to LC3-II. These data show that resveratrol increased the autophagy level of IEC-6 cells after ionizing radiation. After treatment with Ex527, we observed that the expression of both LC3II and beclin-1 and the conversion of LC3-I to LC3-II were inhibited in the cells, indicating that the activation of SIRT1 is required for resveratrol-induced autophagy.

**Fig. 4:**
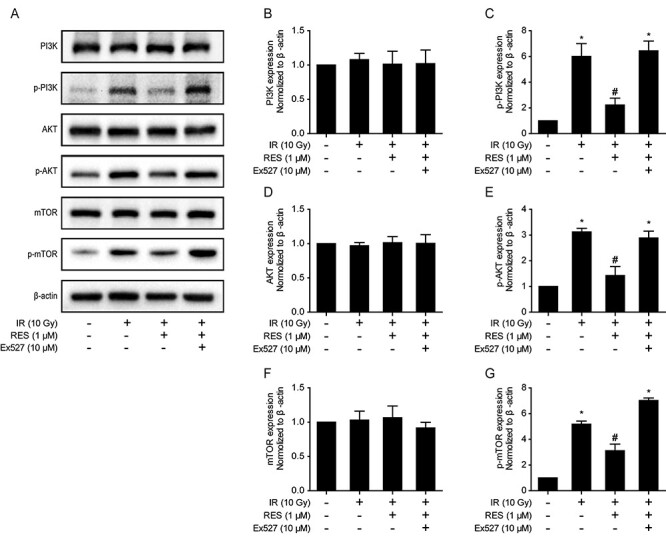
(A) Protein levels of PI3K, p-PI3K, AKT, p-AKT, mTOR and p-mTOR detected by western blots. (B–G) Expression of PI3K, p-PI3K, AKT, p-AKT, mTOR and p-mTOR. Data are expressed as mean ± standard deviation. ^*^P < 0.001 compared with the control group; ^#^*P* < 0.001 compared with the IR group.

We then tested the effect of resveratrol on the PI3K/AKT/mTOR signaling pathway after irradiation. Our results showed that AKT was phosphorylated following PI3K activation, which in turn promoted mTOR phosphorylation, following ionizing radiation; however, pretreating cells with resveratrol reversed these irradiation-induced effects. In contrast, inhibiting SIRT1 with Ex527 increased the levels of p-AKT, mTOR, and p-mTOR, thereby blocking autophagy (Fig. 4).

**Fig. 5.**
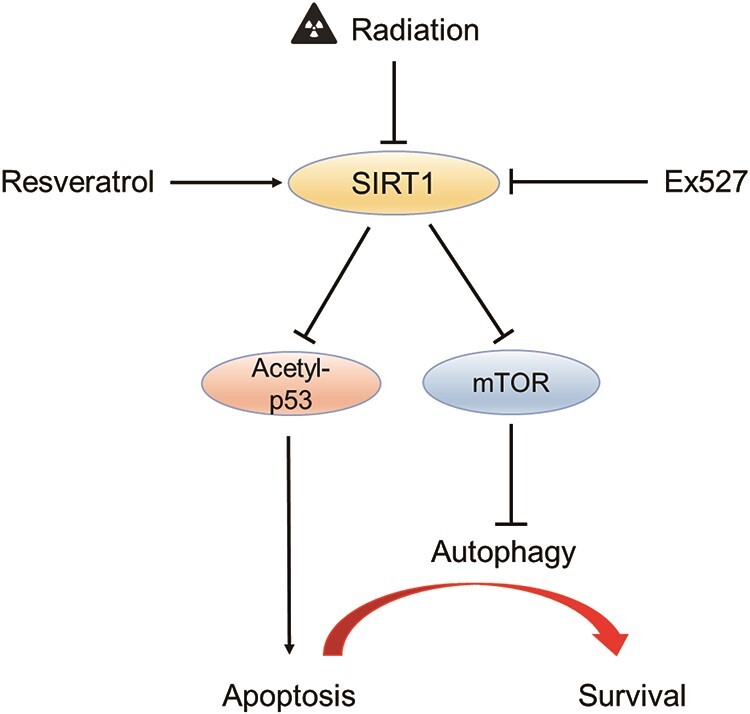
Schematic of the action mechanism of resveratrol in radiation-induced cell damage. Ionizing radiation downregulate the expression of SIRT1, inhibiting autophagy and inducing apoptosis by increasing the mTOR activity and p53 acetylation. Resveratrol mitigates radiation-induced injury by reversing this process, and this protective effect is inhibited by Ex527.

## DISCUSSION

The epithelium of the small intestine is one of the most rapidly renewing tissues and is highly sensitive to irradiation. However, there is still no effective therapeutic measure for radiation-induced small intestinal injury. IEC-6 cells are a normal cell line derived from rat intestinal crypts. It reflects the characteristics of IEC-6 and is generally used as an *in vitro* model of intestinal diseases. In the present study, resveratrol at low concentrations prevented radiation-induced damage in IEC-6 cells by inducing autophagy and inhibiting apoptosis via the SIRT1/mTOR pathway. The mechanism of resveratrol in radiation-induced cell damage is shown in Fig. 5. Beclin-1 and LC3-II are the autophagy markers involved in the formation and maturation of autophagosomes [19]. In our study, ionizing radiation decreased the basal level of autophagy of IEC-6 cells, as indicated by the downregulation of beclin-1 and LC3 -II, specific autophagosome markers [19]. Resveratrol pretreatment increased the level of LC3-II and beclin-1; however, Ex527 treatment reversed this effect compared with the ionizing radiation group. Collectively, these data showed that resveratrol restored autophagy via the activation of SIRT1.

The activity of SIRT1 has been shown to play a critical role in regulating autophagy [20,21]. SIRT1 can regulate autophagy by deacetylating several proteins associated with autophagy, such as Atg5 and Atg7 [21]. Meanwhile, SIRT1 is involved in the regulation of the upstream signaling pathway of autophagy, thereby affecting the initiation of autophagy. In this upstream signaling cascade, SIRT1 induces AMPK phosphorylation and suppresses mTOR phosphorylation to activate autophagy [22]. It has been reported that resveratrol stimulated autophagy via the AMP-activated protein kinase (AMPK)-mTOR pathway [23]. However, our results indicate that the PI3K/mTOR pathway is also involved in resveratrol-induced autophagy and is related to the activation of SIRT1.

The serine/threonine kinase AKT, a downstream target of PI3K, is activated by several stimuli in a PI3K-dependent manner. The PI3K/AKT/mTOR axis is the most characterized autophagy signaling pathway. In this study, resveratrol pretreatment increased SIRT1-induced autophagy in irradiated IEC-6 cells via the PI3K/AKT/mTOR signaling pathway. As shown by western blotting, the levels of p-PI3K and *p*-AKT were markedly increased in the ionizing radiation group, whereas their levels significantly decreased in the resveratrol pretreatment group. mTOR, a negative regulator of autophagy, is positively regulated via PI3K/AKT [24]. In the present study, the expression of *p*-mTOR was significantly increased in the ionizing radiation group, but these levels decreased in the resveratrol pretreatment group. When we used Ex527 to inhibit the SIRT1 activity, the levels of p-PI3K, p-AKT and p-mTOR were downregulated, indicating that resveratrol improved autophagy through the PI3K/AKT/mTOR signaling pathway via enhanced SIRT1 expression and activity.

Furthermore, we studied the effect of resveratrol on radiation-induced apoptosis. The levels of p53 and acetylated p53 were significantly increased in irradiated cells compared with those in the control group. Acetylated p53, the activated form of p53, is more stable than p53 and is required for transcription induction [25]. SIRT1 reduces the transcriptional activity of p53 and suppresses p53-dependent apoptosis and radiosensitivity [26]. In the present study, resveratrol pretreatment inhibited radiation-induced acetylation of p53, and this effect was blocked using Ex527. Bax, a downstream target of p53, is considered a pro-apoptotic protein as it induces the release of cytochrome c from the mitochondria as well as activates caspases-3 and -9, resulting in the induction of apoptosis [27]. Radiation treatment increased the expression of Bax, whereas resveratrol pretreatment reduced this effect. These results indicate that the deacetylation activity of SIRT1 on p53 may be a key factor in modulating the beneficial effects of resveratrol by preventing radiation damage to the small intestine. In addition, there is complex crosstalk signaling between autophagy and p53. However, the mechanisms underlying the reciprocal functional interaction of autophagy-p53 are not yet fully understood. Although p53 induces the transcription of autophagy-related genes, such as *AMPK β1/β2*, *DRAM* and *DAPK-1* [28,29], a previous study suggested that lowering the levels of p53 may facilitate autophagy [30]. In addition, it has been reported that autophagy suppresses p53 expression [31]. In the present study, resveratrol exerted a pleiotropic regulatory effect on p53 and autophagy. Future studies should evaluate whether resveratrol is involved in the interaction between autophagy and p53.

Our study had some limitations. Autophagy is a dynamic process, and different doses of irradiation may influence the biological effects differently. The conclusions drawn from one time point and one radiation dose are tentative. Therefore, the changes in autophagy-related proteins should be further investigated at different time points and different radiation doses. In addition, only one cell line was used in the present study. The protective effect of resveratrol in radiation-induced damage should also be investigated in other intestinal cell lines in the future.

Taken together, our study shows that resveratrol at a low concentration reduces radiation-induced damage in IEC-6 cells by promoting autophagy and inhibiting apoptosis via the SIRT1 activity. Furthermore, suppressing the PI3K/AKT/mTOR signaling pathway resulted in the induction of autophagy. Overall, our study provides evidence that resveratrol could serve as a radiotherapy protectant to improve radiation-induced effects in the intestine.

## FUNDING

This study was supported by the National Natural Science Foundation of China [grant numbers 81972847 and 81573089].

## CONFLICT OF INTEREST

The authors declare no conflict of interest.

## Supplementary Material

Supplementary_FIGURE_1_rrab035

supplementary_materials_rrab035
