# Supplementary figures and images for: Resveratrol protects intestinal epithelial cells against radiation-induced damage by promoting autophagy and inhibiting apoptosis through SIRT1 activation
Source: J Radiat Res. 2021 Apr 29;62(4):574–81. doi: 10.1093/jrr/rrab035 (PMC8273810; doi:10.1093/jrr/rrab035)

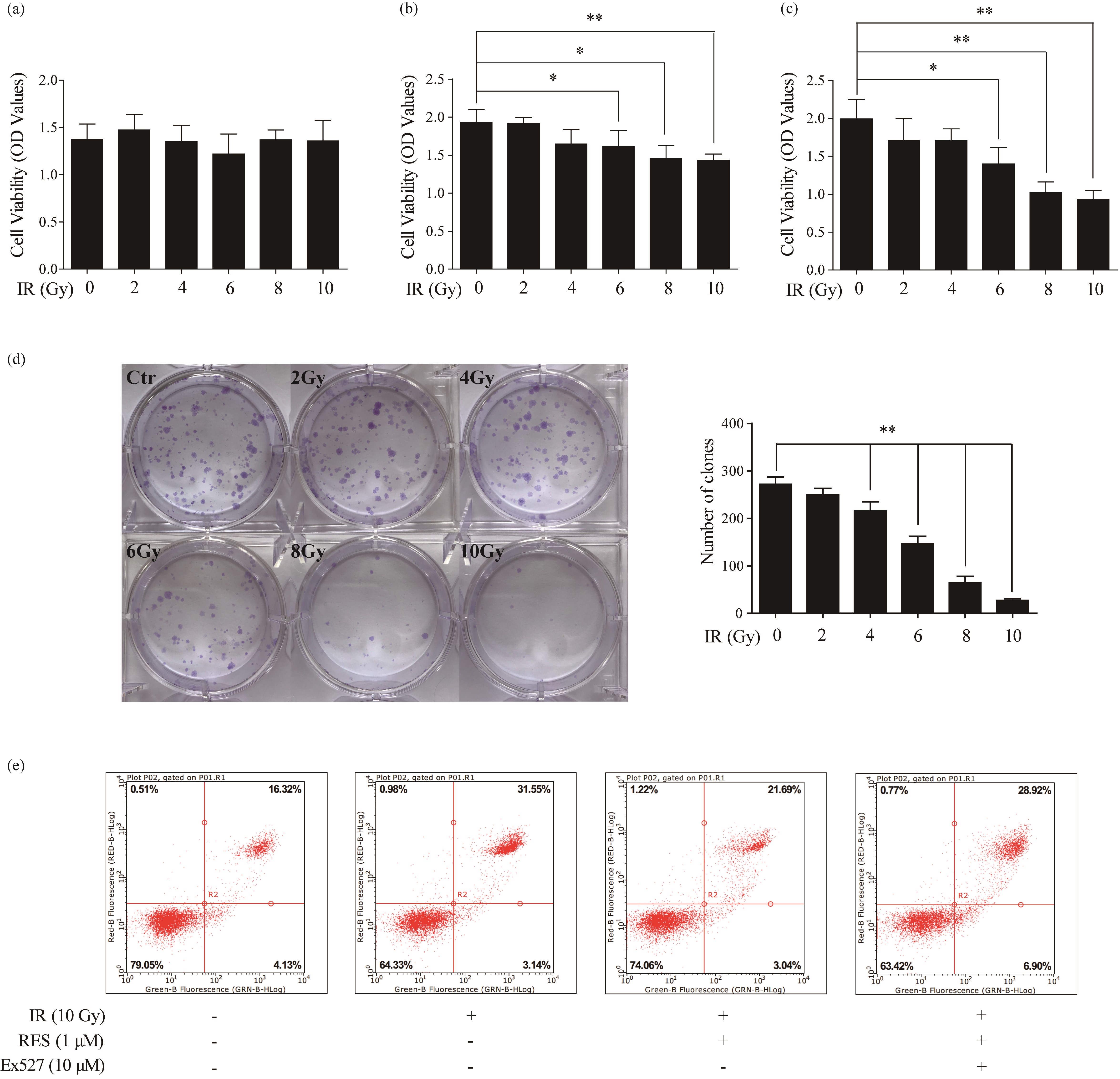

Supplement: Supplementary_FIGURE_1_rrab035 [file supplementary_figure_1_rrab035.jpeg]
