# Supplementary material for: Resveratrol protects intestinal epithelial cells against radiation-induced damage by promoting autophagy and inhibiting apoptosis through SIRT1 activation
Source: J Radiat Res. 2021 Apr 29;62(4):574–81. doi: 10.1093/jrr/rrab035 (PMC8273810; doi:10.1093/jrr/rrab035)
Supplement: supplementary_materials_rrab035 [file supplementary_materials_rrab035.docx]

## Supplementary Materials for

Resveratrol protects intestinal epithelial cells against radiation-induced damage by promoting autophagy and inhibiting apoptosis through SIRT1 activation

**Supplementary materials and methods**

**Cell counting kit 8 (CCK8) assay**

After trypsin digestion (Gibco) of adherent cells, 3000 cells/well were seeded in a 96-well plate (six wells per group). The cells received various doses of ionizing radiation. After irradiation, cells were incubated for 12 h, 24 h and 48 h at 37°C in an atmosphere of 5% CO2. Thereafter, 10 μL of CCK-8 reagent (#CA1210; Solarbio, Beijing, China) was added to each well and incubated for 2 h in dark. Cell viability was determined by measuring the absorbance at 450 nm using a microplate reader (Bio-Rad, Hercules, CA, USA).

**Plate cloning experiment**

After trypsin digestion (Gibco) of adherent cells, 400 cells were seeded per well of a six-well cell culture plate. After 48 h, the cells in each group were treated with different doses of irradiation, followed by cell colony formation in the culture dish for 1-2 weeks in a 37 ℃ cell incubator. The incubation was stopped when a white cell clone was visible. Cells were fixed with 4% paraformaldehyde for 15 min and stained with 0.1% crystal violet for 30 min. Thereafter, images were captured and colonies with diameters > 0.5 mm were enumerated using a microscope (Nikon Eclipse 80i, Tokyo, Japan).

**Result**

**Ionizing radiation inhibits cell viability and proliferation**

The cell viability was detected by CCK-8 assay at 12, 24, and 48 h after irradiation. There was no significant in cell viability between all the group at 12 h after irradiation (Supplementary Figure 1a). However, the viability of cells started to significantly decrease at 24 h after irradiation in a dose-dependent manner, especially when the dose is larger than 6Gy, compared with that of the control (Supplementary Figure 1b). The same trend was also shown in the result of 48 h after irradiation (Supplementary Figure 1c). To validate this result, the cell proliferation was detected by colony formation. The result showed that 10Gy-irradiation markedly suppressed the colony-forming ability of osteosarcoma cells (Supplementary Figure 1d).


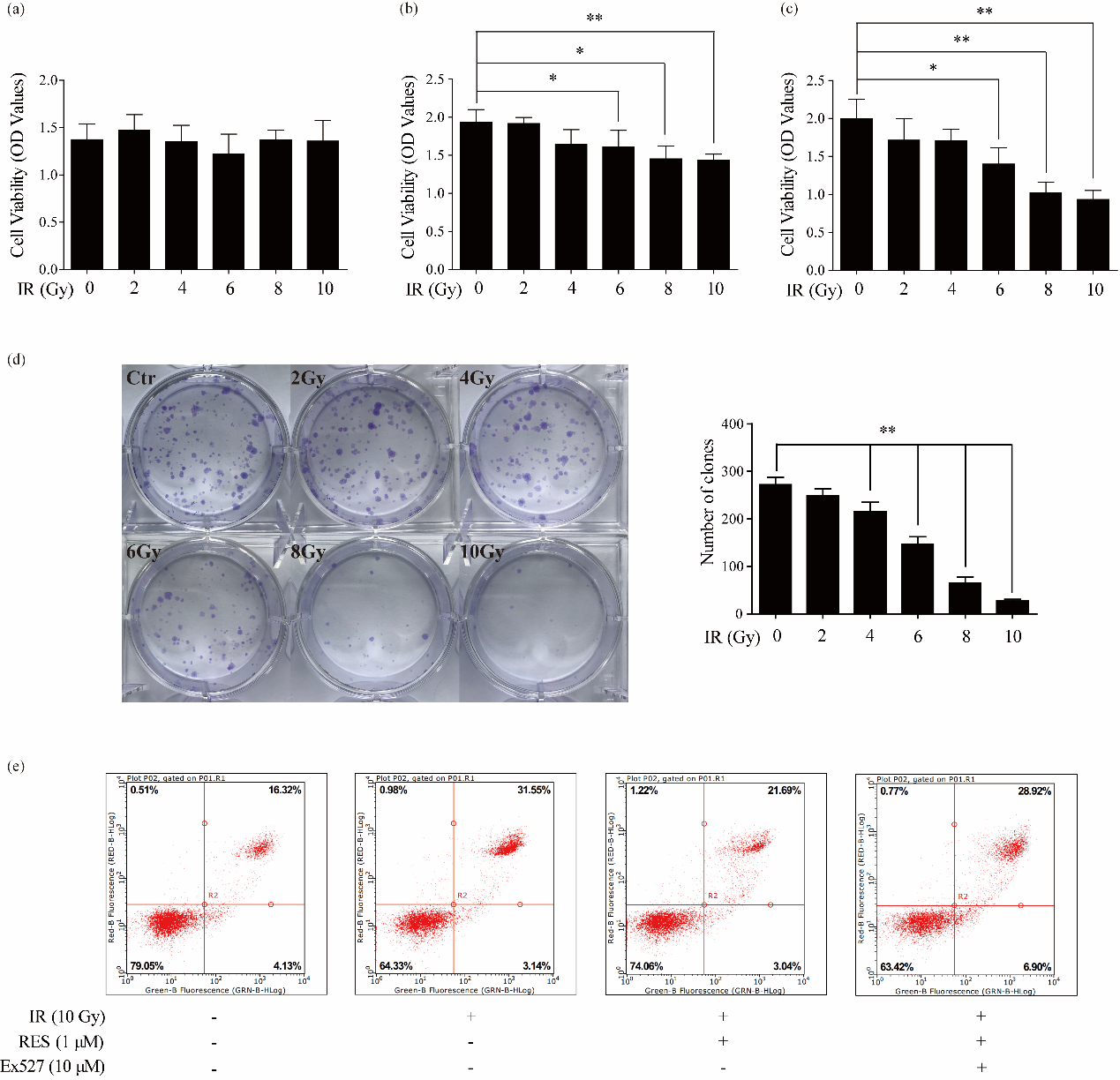


**Supplement Figure 1**. (a) Cell viability was measured using the CCK-8 assay of IEC-6 cells at 12 h after irradiation. (b) Cell viability was measured using the CCK-8 assay of IEC-6 cells at 24 h after irradiation. (c) Cell viability was measured using the CCK-8 assay of IEC-6 cells at 48 h after irradiation. (d) Clone formation ability was evaluated using plate cloning assays. (e) Apoptosis detected using flow cytometry after Annexin V-FITC (RED-B-HLog) and PI (GRN-B-HLog) staining. The percentage of apoptotic cells is indicated by the sum of the numerical values represented in the upper right (annexin+/PI+) and lower right (annexin+/PI-) quadrants. *P < 0.05, **P < 0.01 compared with the Control group. Values are presented as the mean ± SD (n = 3).
